# Supplementary material for: Early Recurrence after Upfront Surgery for Pancreatic Ductal Adenocarcinoma
Source: Curr Oncol. 2023 Mar 27;30(4):3708–20. doi: 10.3390/curroncol30040282 (PMC10137113; doi:10.3390/curroncol30040282)
Supplement: Supplementary file 1 [file curroncol-30-00282-s001.zip › curroncol-2269521-supplementary.pdf]

**Supplementary table S1**. Univariate and multivariate analysis of predictive factors of distant recurrence (for patients with recurrence only).

| | **Univariate analysis** | | **Multivariate analysis** | |
|---|---|---|---|---|
| | **OR (95 IC)** | **p value** | **OR (95 IC)** | **p value** |
| Pre-op CA 19.9 > 70.5 UI/L | 0.94 (0.51-1.75) | 0.852 | | |
| Post-op CA-19.9 > 15 UI/L | 1.26 (0.67-2.39) | 0.472 | | |
| T3-T4 | 1.01 (0.43-2.38) | 0.979 | | |
| Nodal metastases (N+) | 1.25 (0.57-2.77) | 0.580 | | |
| G3-G4 | **2.00 (1.06-3.75)** | **0.031** | 1.82 (0.96-3.46) | 0.067 |
| R1 resection | 1.46 (0.82-2.58) | 0.198 | | |
| Lymphovascular invasion | **1.86 (1.03-3.35)** | **0.039** | 1.70 (0.93-3.10) | 0.083 |
| Perineural invasion | 1.55 (0.45-5.32) | 0.487 | | |

OR: odds ratio; IC: interval confidence; BMI: body mass index; CRP: C-reactive protein; NLR: neutrophil/lymphocyte ratio; PLR: platelet/lymphocyte ratio; PD: Pancreatoduodenectomy; DP: distal pancreatectomy; TP; total pancreatectomy; POPF: post-operative pancreatic fistula.
